# Can the coronavirus disease‐2019 vaccine induce an asthma attack?

**DOI:** 10.1002/ams2.714

**Published:** 2021-11-27

**Authors:** Takaaki Kawasaki, Koichiro Sueyoshi, Yuki Nakamura, Ken Okamoto, Hiroshi Tanaka

**Affiliations:** ^1^ Department of Emergency and Critical Care Medicine Juntendo University Urayasu Hospital Urayasu Chiba Japan

## Funding Information

No funding information provided.

## Disclosures

Approval of Research Protocol: N/A.

Informed Consent: Written informed consent was acquired from the patient’s family.

Registry and the Registration No. of the study/Trial: N/A.

Animal Studies: N/A.

Conflict of Interest: None declared.

Dear Editor,

With the increasing spread of the severe acute respiratory syndrome‐coronavirus‐2 variants worldwide, vaccination remains essential to control the pandemic. However, vaccine hesitancy is common due to concerns about potential side effects. Herein, we present a rare case of fatal asthma, possibly induced by the coronavirus disease‐2019 (COVID‐19) vaccine. A 39‐year‐old female patient with asthma was transferred to our emergency department (ED) due to cardiopulmonary arrest (CPA). She was previously diagnosed with asthma, but was not taking any regular medication. Her last exacerbation occurred 1 year before when she gave birth to twins. Three days after receiving the first dose of the Moderna COVID‐19 (mRNA‐1273) vaccine, she suddenly experienced a severe asthma attack after dinner and the emergency medical services were called. The patient went into CPA, and cardiopulmonary resuscitation was immediately performed by a bystander; this was continued by the emergency medical services personnel until the patient arrived at the ED. Upon arriving at the ED, spontaneous circulation was regained by oxygenation and intravenous administration of 1 mg of adrenaline. Considering the high inspiratory airway pressure after intubation and her medical history, a severe asthma attack was considered as the cause of CPA. The patient was in a vegetative state due to a prolonged CPA (32 min).

Adverse events following immunization, including anaphylaxis, have been reported due to COVID‐19 vaccination. Blumenthal *et al*.^1^ reported that anaphylaxis was confirmed in 9 of 1,365 (0.023%) patients who received the Moderna vaccine. However, asthma attacks triggered by the COVID‐19 vaccine have not yet been reported. According to a report by the Centers for Disease Control and Prevention, the United States Food and Drug Administration has identified minor adverse events, including myocardial infarction, cholecystitis, and nephrolithiasis, as being possibly related to the vaccines.^2^ As per this report, one case of intractable nausea/vomiting and two cases of facial swelling in individuals with a previous history of cosmetic filler injections were related to the vaccine. The report further states that the role of the vaccine in the development of adverse events, including rheumatoid arthritis, peripheral edema/dyspnea with exertion, and autonomic dysfunction, cannot be excluded.^2^ Likewise, in the present case, the role of vaccination in the development of asthma attacks was not excluded. Although, to our best knowledge, there is no evidence on the increased risk of asthma attacks in patients with asthma who received COVID‐19 vaccination, the fever or illnesses induced by the vaccine can trigger asthma attacks. Therefore, patients with asthma should be reviewed for optimal disease control before vaccination.
